# Anti-Signal Recognition Particle Antibody-Associated Severe Interstitial Lung Disease Requiring Lung Transplantation

**DOI:** 10.7759/cureus.7962

**Published:** 2020-05-05

**Authors:** Anam Qureshi, Daniel Brown, Lawrence Brent

**Affiliations:** 1 Internal Medicine, Reading Hospital - Tower Health, West Reading, USA; 2 Pathology and Laboratory Medicine, Lehigh Valley Health Network, Allentown, USA; 3 Rheumatology, Temple University Hospital, Philadelphia, USA

**Keywords:** anti-signal recognition particle, myopathy, interstitial lung disease, lung transplantation

## Abstract

Anti-signal recognition particle (SRP) antibodies are typically associated with immune-mediated necrotizing myopathy. Some patients with anti-SRP antibodies may have extramuscular manifestations including mild respiratory symptoms secondary to interstitial lung disease. We present a case of a 40-year-old female presenting with acute hypoxic respiratory failure secondary to anti-SRP antibody-associated interstitial lung disease with mildly elevated creatinine kinase but without evidence of necrotizing myopathy on muscle biopsy. After a complicated six-month hospitalization, the patient successfully received double lung transplantation and was eventually discharged on room air. Unexplained worsening interstitial infiltrates leading to persistent hypoxic respiratory failure in the setting of nonspecifically elevated creatinine kinase should warrant consideration of an underlying connective tissue disease, including myositis with anti-SRP antibody-associated interstitial lung disease. In rare cases, interstitial lung disease may be severe requiring lung transplantation.

## Introduction

Signal recognition particle (SRP) assists in the transfer of newly generated proteins through the endoplasmic reticulum [1]. Anti-SRP antibodies, first reported by Reeves et al, are typically associated with severe muscular weakness secondary to immune-mediated necrotizing myopathy (IMNM) [1-3]. Extramuscular manifestations may be seen in some patients with anti-SRP antibodies, including pulmonary involvement (14%) [4]. The most common pulmonary manifestation in patients with anti-SRP antibodies is interstitial lung disease (ILD) which usually presents with mild respiratory symptoms and nonspecific interstitial fibrosis on imaging [1]. In patients with anti-SRP antibody-associated inflammatory myopathy, the presence of ILD has been associated with better outcomes [1]. We present an atypical case of anti-SRP antibody-associated ILD causing severe hypoxic respiratory failure requiring lung transplantation, associated with mild myopathy. 

## Case presentation

**Figure 1:**
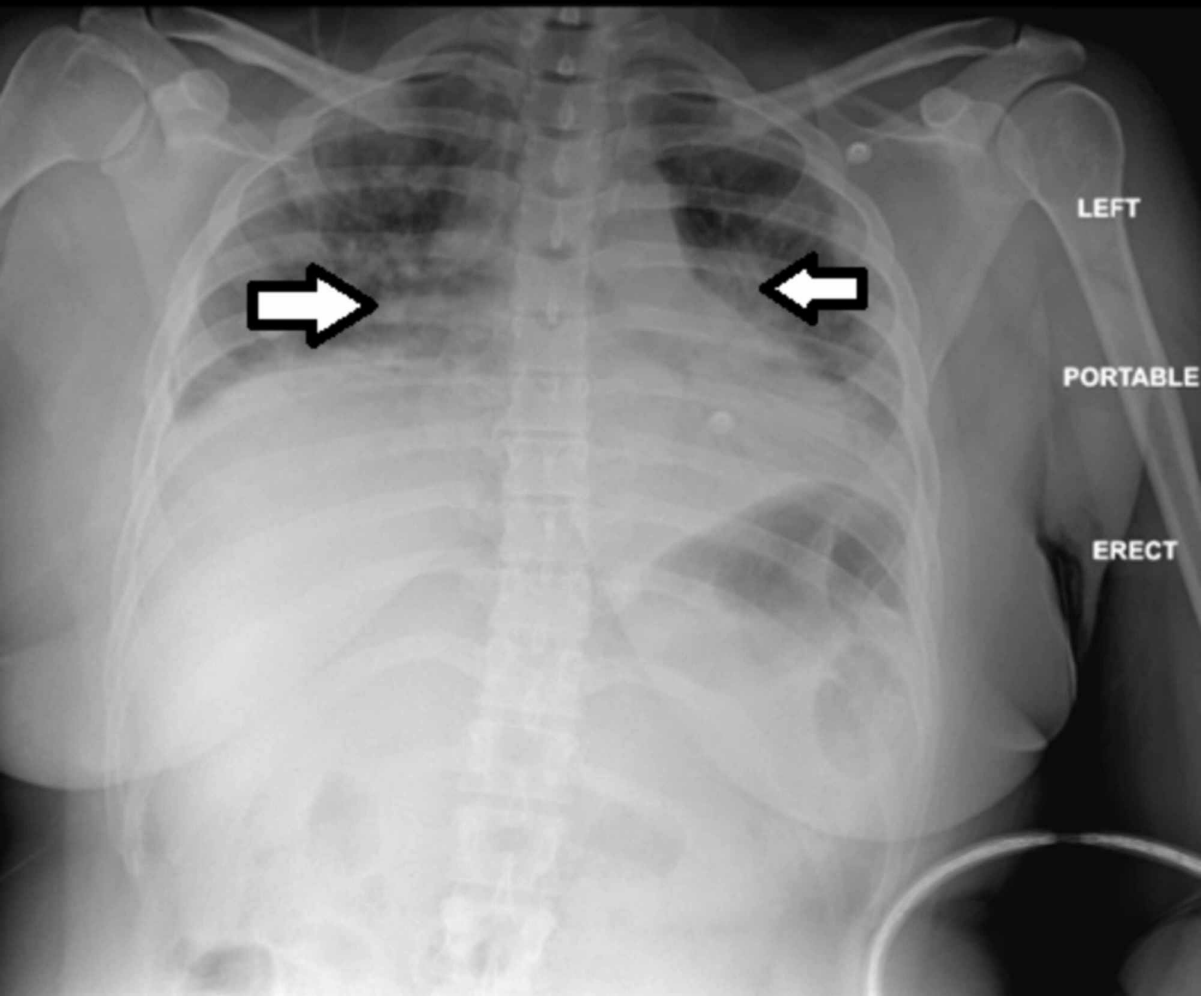
Chest X-ray of the patient at presentation to the emergency department showing low lung volumes, vascular crowding (see arrows), and bibasilar consolidations.

A 40-year-old African American female presented to the emergency department with progressive shortness of breath. She had been diagnosed with community-acquired pneumonia three months prior and had failed outpatient treatments with azithromycin and doxycycline, eventually requiring hospitalization. Her respiratory symptoms recurred shortly after the recent hospitalization. She denied any muscle weakness. On examination, she appeared in mild respiratory distress, was hypoxic with oxygen saturation of 72%, and required high flow oxygen. Her vital signs were as follows: blood pressure 144/94 mmHg, pulse 110/min, respiratory rate 24/min, and temperature 98.3°F. On pulmonary examination, rales were heard bilaterally. She had a nonfocal neurological examination without any motor deficits. No cutaneous ulcerations and arthropathy were noted. Rest of the physical examination was unremarkable. Laboratory studies revealed a white blood cell count of 5,100/µL, an erythrocyte sedimentation rate of 32 mm/hr, and C-reactive protein of 7.6 mg/dL. The serum creatinine kinase (CK) was slightly elevated at 1,303 U/L. Chest X-ray showed low lung volumes, vascular crowding, and worsening bibasilar consolidations compared to the prior imaging (Figure 1). 

**Figure 2:**
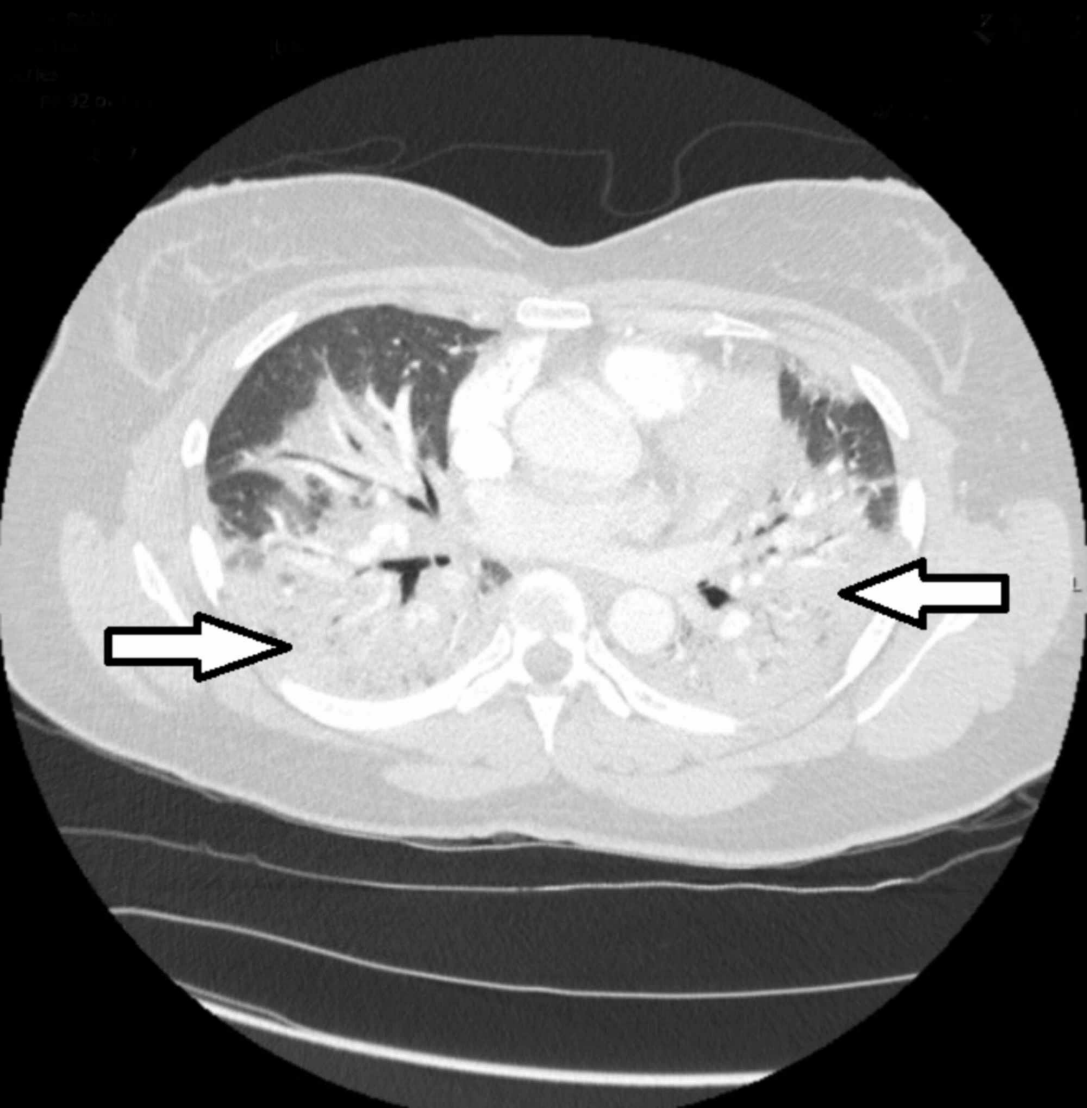
Computerized tomography of the chest at presentation showing extensive multifocal areas of consolidation in the lungs bilaterally (arrows).

Computerized tomography (CT) of the chest showed extensive multifocal areas of consolidation and large pleural effusions without evidence of pulmonary embolism (Figure 2). 

**Figure 3:**
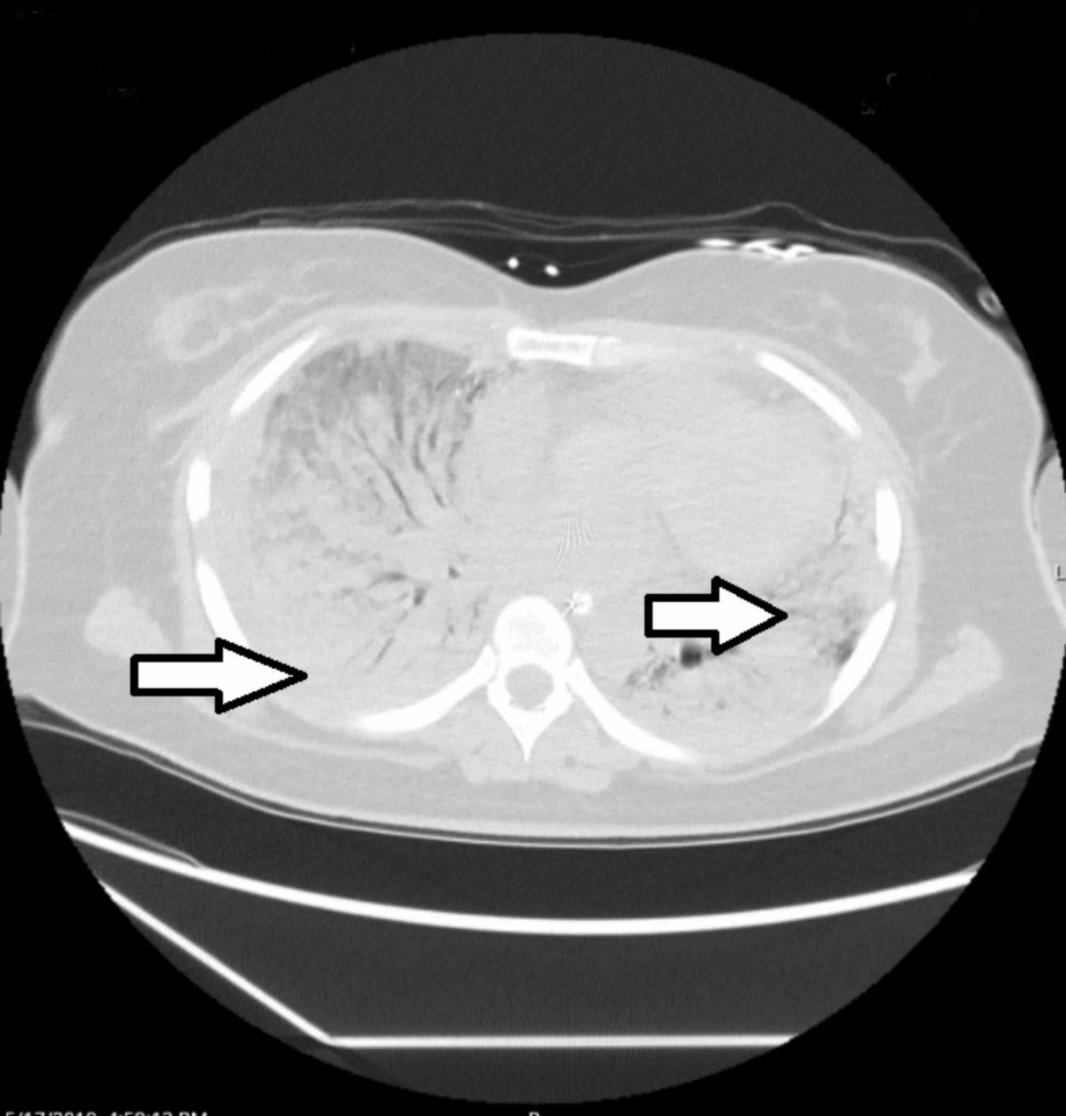
Computerized tomography of the chest 17 days after admission showing worsening ground-glass opacities and consolidative changes of the lungs bilaterally (arrows).

The infectious work-up including sputum and blood cultures was negative. Over the next two days, she developed worsening hypoxic respiratory failure despite being on broad-spectrum antibiotics requiring mechanical ventilation. By day 10, she was dependent on extracorporeal membrane oxygenation. On day 17, CT of the chest showed near complete involvement of the lungs with mixed ground-glass opacities and consolidative changes (Figure 3).

**Figure 4:**
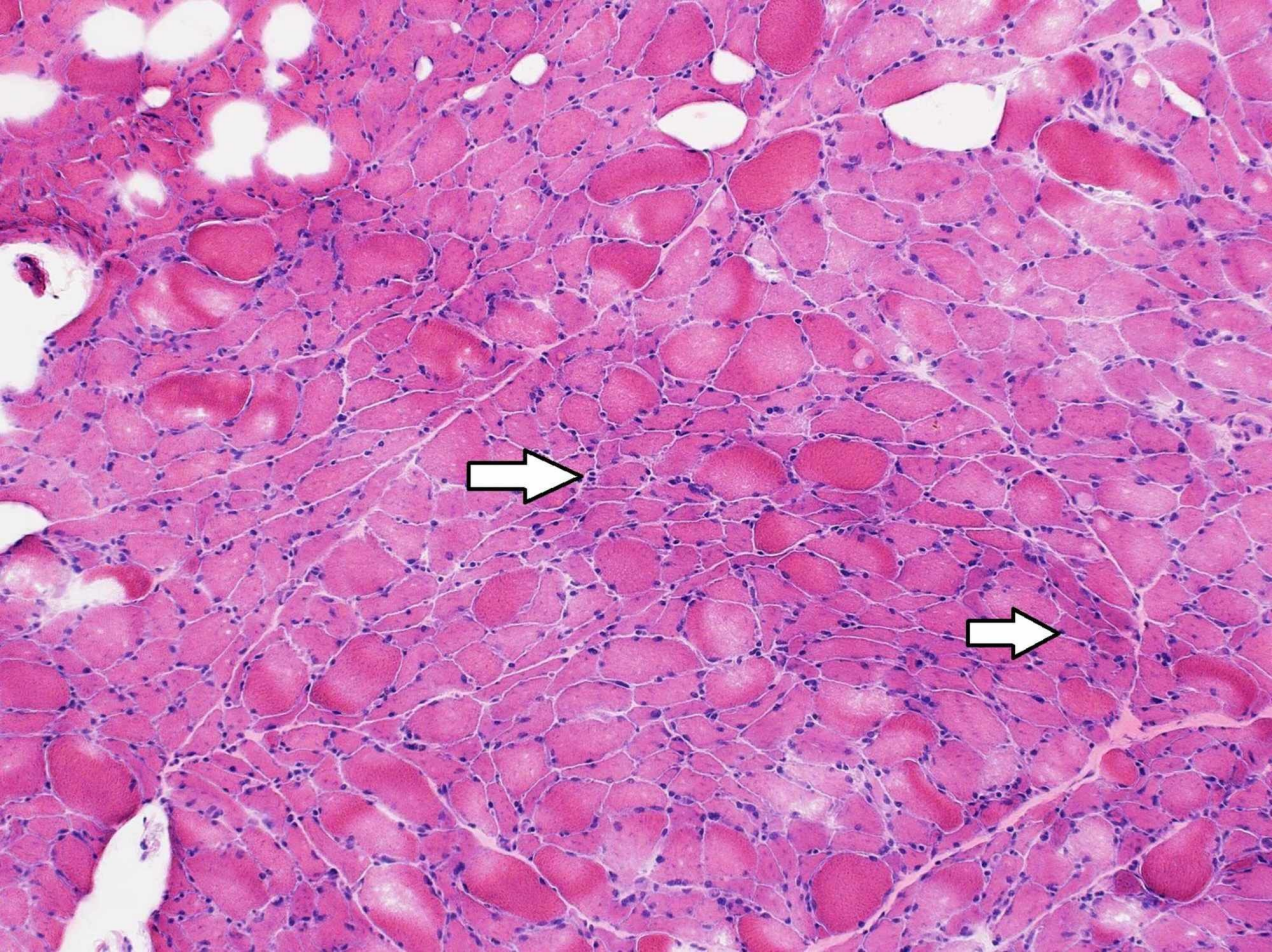
Hematoxylin and eosin stain of the left thigh muscle biopsy showing several angular atrophic fibers, with no myopathic features and no evidence of inflammatory cell infiltrates (original magnification 100x) (arrows).

**Figure 5:**
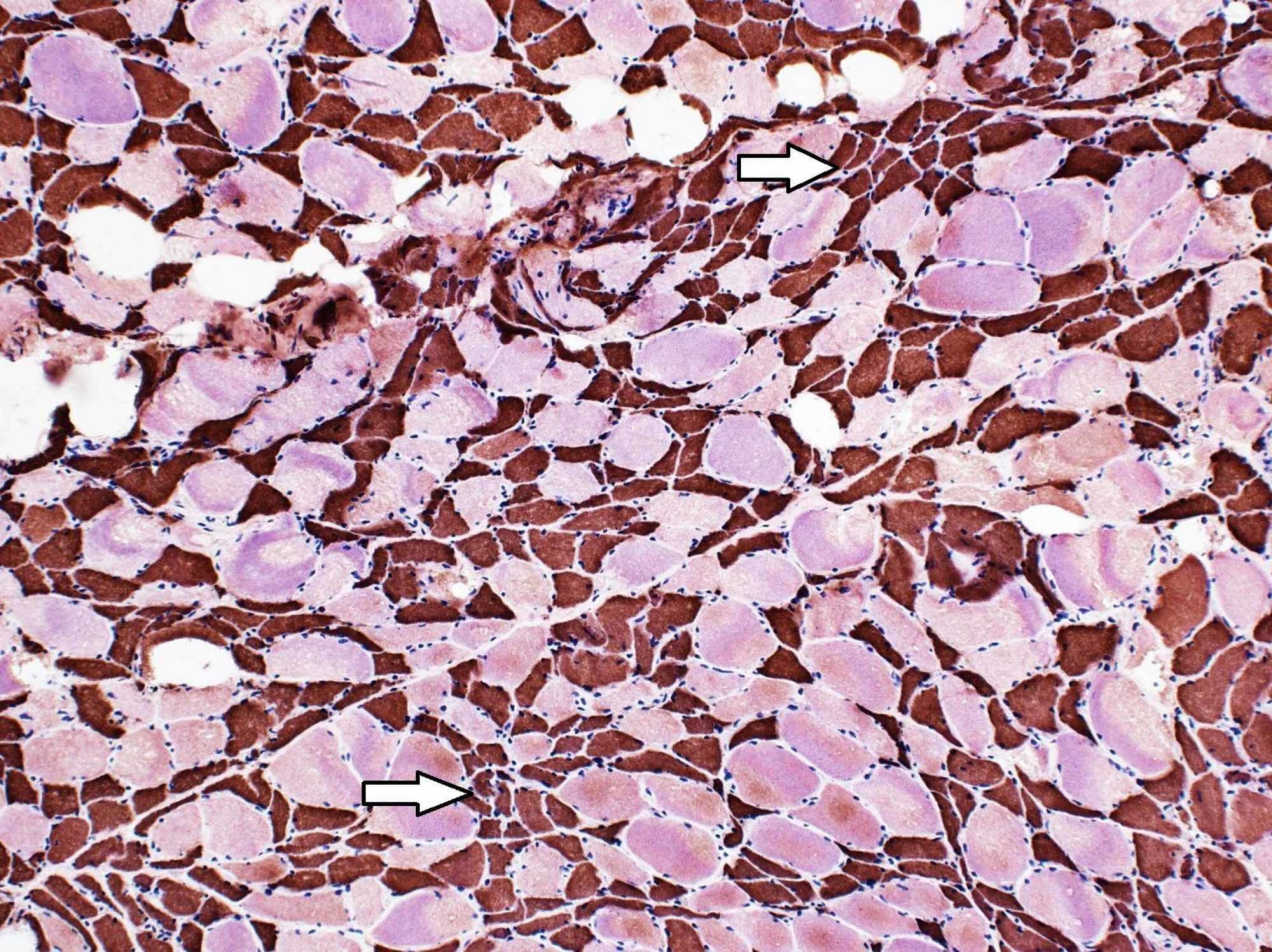
The fast myosin heavy chain of the left thigh muscle biopsy staining the atrophic muscle fibers brown indicative of type II muscle fibers (type I fibers stain pink/red) (original magnification 100x) (arrows).

**Figure 6:**
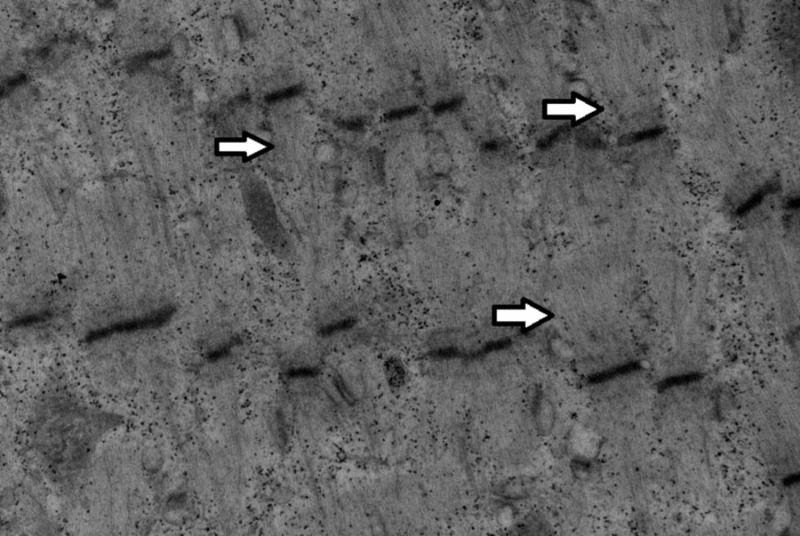
High-power electron microscopy image showing an abnormal left thigh muscle fiber with loss of A band characteristic of critical illness myopathy (arrows).

The patient required tracheostomy to continue mechanical ventilation. Her comprehensive laboratory work-up showed normal thyroid function tests, complement levels, and negative anti-nuclear antibody, anti-Smith, anti-double-stranded DNA, rheumatoid factor, anti-cyclic citrullinated peptide, anti-centromere, anti-Scl 70 (topoisomerase 1), anti-nuclear ribonucleoprotein, anti-Sjögren Syndrome A (SSA), anti-SSB, anti-neutrophil cytoplasmic antibodies, anti-Jo-1, anti-PL7, anti-PL12, anti-EJ, anti-OJ, anti-Mi2, and anti-Ku. However, anti-SRP antibodies were detected (> 11 S.I. using line blot immunoassay). During the course of her hospitalization, she developed severe generalized weakness. She underwent left thigh muscle biopsy showing critical illness myopathy without evidence of necrotizing myositis (Figures 4-6). 

She was treated with methylprednisolone for suspected anti-SRP related severe ILD and mild myopathy with subsequent normalization of CK to 224 U/L but without any improvement of hypoxic respiratory failure. She then received rituximab 1,000 mg (two doses two weeks apart), followed by cyclophosphamide (one dose of 750 mg/m^2^) with persistent ventilator dependent hypoxic respiratory failure. After a prolonged six-month hospitalization, she underwent double lung transplantation. Her postoperative course was complicated by Enterobacter pneumonia and Staphylococcus epidermidis bacteremia. She responded to appropriate antibiotics, and was discharged on room air nearly two months after lung transplant on tacrolimus, mycophenolate mofetil, and prednisone. By the time of discharge, patient’s generalized weakness had improved, and she was able to ambulate. One-month after discharge, the patient was doing well without any evidence of lung transplant rejection or myopathy.

## Discussion

Anti-SRP antibodies, predominantly seen in the Caucasians, are the most common antibodies associated with IMNM [3,4]. Over half of the patients with IMNM have anti-SRP antibodies [3]. Antibodies against HMG-coenzyme A reductase (anti-HMGCR) are also associated with IMNM in about 25% of such patients [5]. The presence of anti-SRP antibodies is usually indicative of IMNM [1,6]. In a study by Suzuki et al., 84% of the patients with inflammatory myopathy and anti-SRP antibodies had IMNM on histological evaluation [1]. Patients with anti-SRP antibody-associated myopathy typically have markedly elevated CK levels, with median CK levels of about 8,000 U/L [4]. We described an unusual case of an African American female presenting with anti-SRP antibody-associated severe ILD causing ventilator-dependent respiratory failure, but only had mildly elevated CK level without evidence of necrotizing myopathy.

Extramuscular manifestations may be seen in patients with anti-SRP antibody-associated myopathy. In a study of 100 patients with inflammatory myopathy and anti-SRP antibodies, 13 patients had ILD [1]. These patients with anti-SRP antibody-associated ILD had mild respiratory symptoms and nonspecific interstitial fibrosis on CT of the chest [1]. Anti-SRP antibodies are more commonly associated with respiratory insufficiency than anti-HMGCR antibodies [7]. In atypical cases, patients may have anti-SRP antibody-associated ILD without myopathy, or develop myopathy at a later time [8,9]. Our case suggests that in patients with recurrent unexplained respiratory symptoms and worsening pulmonary interstitial infiltrates not responding to antibiotics, physicians should consider connective tissue disease-associated ILD, including anti-SRP-positive myositis even in the absence of markedly elevated CK levels and necrotizing myositis. Most patients with anti-SRP antibody-associated IMNM experience improvement of symptoms with corticosteroids and immunosuppression; the pulmonary manifestations are typically mild [4]. However, prompt recognition is important as rarely patients may have severe hypoxic respiratory failure from anti-SRP antibody-associated ILD and lung transplantation may be lifesaving.

## Conclusions

Anti-SRP antibodies are typically associated with necrotizing myopathy with or without mild respiratory symptoms. However, in patients with unexplained acute hypoxic respiratory failure with worsening interstitial infiltrates and mildly elevated CK levels, clinicians should consider atypical presentation of anti-SRP antibody-associated ILD. Rarely, anti-SRP antibodies may be associated with severe lung disease that may require lung transplantation.
